# Supplementary material for: Unsupervised generative and graph representation learning for modelling cell differentiation
Source: Sci Rep. 2020 Jun 17;10:9790. doi: 10.1038/s41598-020-66166-8 (PMC7300092; doi:10.1038/s41598-020-66166-8)
Supplement: Supplementary file 1 — Supplementary materials. [file 41598_2020_66166_MOESM1_ESM.pdf]

Supplementary materials: Unsupervised generative and graph representation learning for modelling cell differentiation

Ioana Bica[1, 4, +, *], Helena Andrés-Terré[2, *], Ana Cvejic[3], and Pietro Liò[2]

[1]Department of Engineering Science, University of Oxford, Oxford, OX1 3PJ, United Kingdom
[2]Department of Computer Science and Technology, University of Cambridge, Cambridge, CB3 0FD, United Kingdom
[3]Wellcome Trust Sanger Institute, Wellcome Trust Genome Campus, Cambridge, CB10 1SA, UK
[4]The Alan Turing Institute, London, NW1 2DB, United Kingdom
[+]Work done while part of the Department of Computer Science and Technology at the University of Cambridge.
[*]these authors contributed equally to this work

Results on dataset with human pancreatic cells

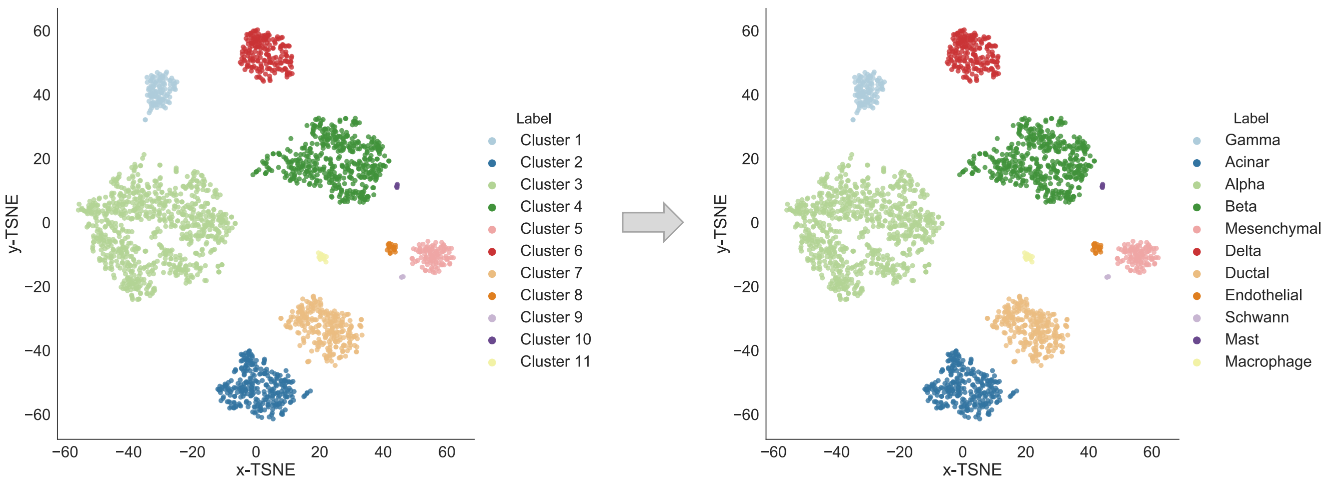

Figure 1. Clusters identified by DiffVAE in the dataset with human pancreatic cells.

Cluster 1 (Gamma)	Cluster 2 (Acinar)	Cluster 3 (Alpha)	Cluster 4 (Beta)	Cluster 5 (Mesenchymal)	Cluster 6 (Delta)
PPY, SERTM1, CARTPT, LMO3, ZNF503	PRSS1[1], REG1B[1], ALB[1]	GCG[1], LOXL4[1], CRYBA2[1], IRX2, FEV[1,2]	MAFA[1], INS[1], SIX2[1],	COL1A1[1], COL1A2[1], COL3A1[1], SPARC[1]	SST[1], RBP4[1], GABRG2[1], GHSR[1]
**Cluster 7 (Ductal)**	**Cluster 8 (Endothelial)**	**Cluster 9 (Schwann)**	**Cluster 10 (Mast)**	**Cluster 11 (Macrophage)**	
KRT19[1], SPP1[1],	SOX18[1], SNAI1[1], BCL6B[1]	NPY, GFRA3[3]	HDC[4], TPSAB1[5], KIT[6,7]	C3AR1[8], CD300A[9], STAB1[10]	

Table 1. High weight genes computed using the high weight connections to the latent dimensions with the highest percentage for differentiating the corresponding cell type. Using references from scientific literature each cluster found using DiffVAE in the dataset with human pancreatic cells is mapped to a cell type.

		Latent representation				T-SNE embedding of latent representation			
Clustering method	Dim size (m)	**DiffVAE**	**VAE**	**AE**	**PCA**	**DiffVAE**	**VAE**	**AE**	**PCA**
**k-means**	20	**0.678**	0.605	0.527	0.549	0.683	0.689	**0.697**	0.689
	50	**0.636**	0.612	0.525	0.283	**0.697**	0.681	0.694	0.654
	100	0.607	0.605	**0.633**	0.557	**0.706**	0.686	0.676	0.658
**DBSCAN**	20	0.020	0.0001	0.021	0.002	**0.932**	0.927	0.887	0.837
	50	0.292	0.001	0.073	0.008	**0.933**	0.891	0.856	0.865
	100	0.345	0.021	0.0005	0.042	**0.957**	0.943	0.867	0.853

Table 2. Human pancreatic cells. Mean ARI obtained for clustering the latent representation and the t-SNE embedding of the latent representation for three setting of the reduced dimension size m.

Results on dataset with human hematopoietic cells

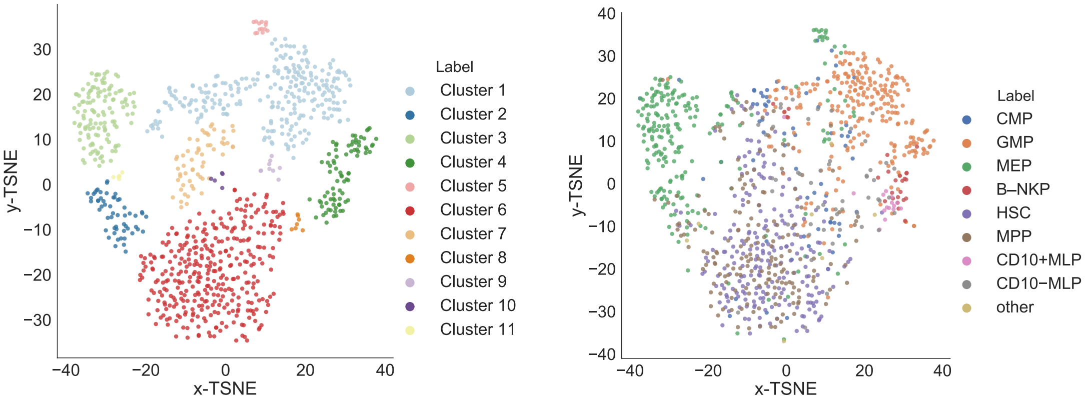

(a) Clusters identified using DiffVAE

(b) Cell labels reported in Velten et al.[11]

Figure 2

As it can be noticed in Supplementary Figure 2, the latent representation obtained through DiffVAE does not produce well-defined cell clusters for the dataset with human hematopoietic cells. Supplementary Figure 2 (a) shows the clusters obtained when using DBSCAN on the T-SNE embedding computed on top of the 50-dimensional representation obtained through DiffVAE. The high weight genes for Cluster 2 are GATA2, GATA1, RRM2, ITGA2B, MYBL2 and for Cluster 3 are GATA1, TYMS, KLF1, TFR2 which helps us determine that Clusters 2 and 3 contain the MEP cells. High weight genes for cluster 1 include: CTSG, AZU1, ELANE, LYZ, ELANE, LGMN indicates that the GMP cells are part of this cluster. Moreover, the presence of B cells in cluster 4 is indicated by some of the high weight genes for cluster 4: DNTT, VPREB1, JCHAIN.

Nevertheless, in Supplementary Figure 2 (b) we plotted the cell labels (based on FACS surface phenotype) provided by Velten et al.[11]. We notice that the HSC and MPP cells cluster together. Similarly, the CMP cells are also scattered and do not form a cluster. Velten et al.[11] reported similar results when applying clustering methods to this dataset.

The limitations of DiffVAE for this dataset may be caused by a large number of different cell states compared to the number of samples available to distinguish between them. This problem is also amplified by the fact that the cell states are close to each other in the process of haematopoiesis.

References

1. Muraro, M. J. *et al.* A single-cell transcriptome atlas of the human pancreas. *Cell systems* **3**, 385–394 (2016).
2. Kimple, M. E. *et al.* Prostaglandin e2 receptor, ep3, is induced in diabetic islets and negatively regulates glucose-and hormone-stimulated insulin secretion. *Diabetes* **62**, 1904–1912 (2013).
3. Widenfalk, J., Tomac, A., Lindqvist, E., Hoffer, B. & Olson, L. Gfrα-3, a protein related to gfrα-1, is expressed in developing peripheral neurons and ensheathing cells. *Eur. journal neuroscience* **10**, 1508–1517 (1998).
4. Kuramasu, A., Saito, H., Suzuki, S., Watanabe, T. & Ohtsu, H. Mast cell-/basophil-specific transcriptional regulation of human l-histidine decarboxylase gene by cpg methylation in the promoter region. *J. Biol. Chem.* **273**, 31607–31614 (1998).
5. Caughey, G. H. Mast cell tryptases and chymases in inflammation and host defense. *Immunol. reviews* **217**, 141–154 (2007).
6. Garcia-Montero, A. C. *et al.* Kit mutation in mast cells and other bone marrow hematopoietic cell lineages in systemic mast cell disorders: a prospective study of the spanish network on mastocytosis (rema) in a series of 113 patients. *Blood* **108**, 2366–2372 (2006).
7. Cruse, G., Metcalfe, D. D. & Olivera, A. Functional deregulation of kit: link to mast cell proliferative diseases and other neoplasms. *Immunol. Allergy Clin.* **34**, 219–237 (2014).

8. Mamane, Y. *et al.* The c3a anaphylatoxin receptor is a key mediator of insulin resistance and functions by modulating adipose tissue macrophage infiltration and activation. *Diabetes* **58**, 2006–2017 (2009).

9. Zenarruzabeitia, O., Vitallé, J., Eguizabal, C., Simhadri, V. R. & Borrego, F. The biology and disease relevance of cd300a, an inhibitory receptor for phosphatidylserine and phosphatidylethanolamine. *The J. Immunol.* **194**, 5053–5060 (2015).

10. Rantakari, P. *et al.* Stabilin-1 expression defines a subset of macrophages that mediate tissue homeostasis and prevent fibrosis in chronic liver injury. *Proc. Natl. Acad. Sci.* **113**, 9298–9303 (2016).

11. Velten, L. *et al.* Human haematopoietic stem cell lineage commitment is a continuous process. *Nat. cell biology* **19**, 271 (2017).
